# Antibiotic exposure and the development of coeliac disease: a nationwide case–control study

**DOI:** 10.1186/1471-230X-13-109

**Published:** 2013-07-08

**Authors:** Karl Mårild, Weimin Ye, Benjamin Lebwohl, Peter HR Green, Martin J Blaser, Tim Card, Jonas F Ludvigsson

**Affiliations:** 1 Clinical Epidemiology Unit, Karolinska Institutet, Stockholm, Sweden; 2 Astrid Lindgren Children’s Hospital, Solna, Sweden; 3 Department of Medical Epidemiology & Biostatistics, Karolinska Institutet, Stockholm, Sweden; 4 Celiac Disease Center, Department of Medicine, Columbia University Medical Center, Columbia University, New York, USA; 5 Department of Medicine, New York University Langone Medical Center, New York, USA; 6 Division of Epidemiology and Public Health, University of Nottingham, Nottingham City Hospital, Nottingham, UK; 7 Department of Paediatrics, Örebro University Hospital, Örebro, Sweden

**Keywords:** Celiac, Inflammation, Microbiota, Population-based case–control study

## Abstract

**Background:**

The intestinal microbiota has been proposed to play a pathogenic role in coeliac disease (CD). Although antibiotics are common environmental factors with a profound impact on intestinal microbiota, data on antibiotic use as a risk factor for subsequent CD development are scarce.

**Methods:**

In this population-based case–control study we linked nationwide histopathology data on 2,933 individuals with CD (Marsh stage 3; villous atrophy) to the Swedish Prescribed Drug Register to examine the association between use of systemic antibiotics and subsequent CD. We also examined the association between antibiotic use in 2,118 individuals with inflammation (Marsh 1–2) and in 620 individuals with normal mucosa (Marsh 0) but positive CD serology. All individuals undergoing biopsy were matched for age and sex with 28,262 controls from the population.

**Results:**

Antibiotic use was associated with CD (Odds ratio [OR] = 1.40; 95% confidence interval [CI] = 1.27-1.53), inflammation (OR = 1.90; 95% CI = 1.72–2.10) and normal mucosa with positive CD serology (OR = 1.58; 95% CI = 1.30–1.92). ORs for prior antibiotic use in CD were similar when we excluded antibiotic use in the last year (OR = 1.30; 95% CI = 1.08-1.56) or restricted to individuals without comorbidity (OR = 1.30; 95% CI = 1.16 – 1.46).

**Conclusions:**

The positive association between antibiotic use and subsequent CD but also with lesions that may represent early CD suggests that intestinal dysbiosis may play a role in the pathogenesis of CD. However, non-causal explanations for this positive association cannot be excluded.

## Background

Coeliac disease (CD) is a life-long autoimmune disease prevalent in 1 to 2% of the western population [1]. CD is a multifactorial disease where genetically predisposed individuals develop small-intestinal villous atrophy and inflammation in response to dietary gluten intake [2]. In recent decades, the prevalence of CD has more than doubled, [3] strongly indicating that environmental factors other than gluten-exposure may have a significant influence on CD development [4]. Further, data from the “Swedish celiac epidemic”, where childhood CD incidence displayed an epidemic pattern with a rapid four-fold increase in incidence in 1984 and a later abrupt decline in 1996, coinciding with changed infant feeding recommendations, have suggested that environmental factors influence CD development [5].

Today, half of all children in many Western countries receive antibiotics at least once a year [6]. Antibiotics can have both short- and long-term effects on the ecological balance between the host and the normal microbiota [7,8]. The intestinal microbiota influences the development of the intestinal immune system, the establishment of oral tolerance and the mucosal barrier function [9]. Previous research has found a difference in the gut microbiota between individuals with CD and healthy controls, suggesting that a dysbiotic microbiota may play a pathogenic role in CD [10]. Despite the profound impact of antibiotics on the gut microbiome, there are few data on antibiotic exposure and risk of CD.

The main objective of this case–control study was to examine the association between antibiotic use and subsequent CD by comparing individuals with CD with matched controls from the general population. We also examined antibiotic use in individuals who may have early CD without villous atrophy [11] (I) small-intestinal inflammation without villous atrophy, or (II) normal small-intestinal mucosa but positive CD serology. Studying these early CD manifestations may be important because risk factors may not only influence the fully developed disease, but sometimes have an even stronger association with disease precursors. For example, cigarette smoking has been more strongly associated with colorectal adenomas compared with colorectal cancer [12,13].

## Methods

In this case–control study we linked nationwide histopathology data on individuals undergoing small intestinal biopsy to the Swedish Prescribed Drug Register in order to examine the association between use of antibiotics and CD. We hypothesized a positive association between antibiotic use and CD.

### Literature search

A literature search at PubMed (http://pubmed.gov/) was performed using the following combinations of words as our major search terms: “celiac”, “coeliac”, “antibiotic” and “antimicrobial”.

### Study population

Between 2006 and 2008, we searched the computerized register of Sweden’s 28 pathology departments to identify individuals with CD [14]. In this study CD was defined as small-intestinal villous atrophy (Marsh grade 3) [15]. An earlier evaluation has shown that 95% of Swedish individuals with villous atrophy have CD [14]. To examine the context of the association between antibiotic use and subsequent CD we also identified individuals with small-intestinal inflammation (Marsh grade 1–2) but without villous atrophy and individuals with normal small-intestinal mucosa (Marsh grade 0) but with positive CD serology [16]. The biopsies were performed between July 1969 and January 2008 [17]. A detailed account of the data collection process has been described elsewhere [14,16].

In the current study we used the same dataset described in our previous study of mortality (29,096 individuals with CD, 13,306 individuals with inflammation, 3,719 individuals with normal mucosa but positive CD serology) [18]. Data on individuals with normal mucosa and positive CD serology were regional and obtained from the ascertainment areas of eight Swedish university hospitals covering approximately half of the Swedish population [16]. Positive CD serology was defined as a positive IgA or IgG AGA (antigliadin), EMA (endomysial), or TTG (tissue transglutaminase) test less than 180 days before or no later than 30 days after a normal biopsy (and with no prior or subsequent biopsy showing villous atrophy or inflammation) [16]. In a recent consensus paper individuals with normal mucosa and positive CD serology were identified as having potential CD [11].

**Figure 1:**
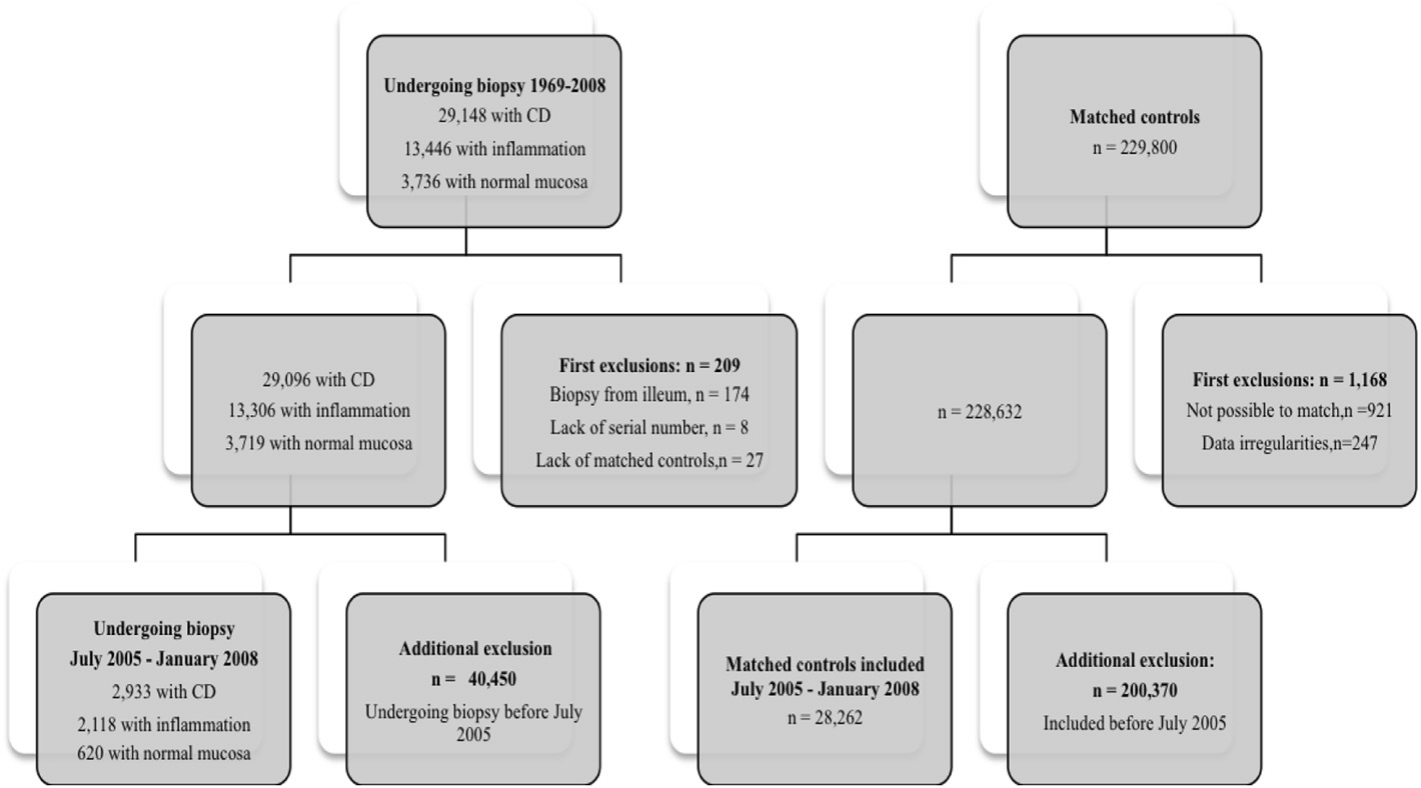
**Flow chart of exclusion criteria.** CD, Coeliac disease.

For each individual undergoing biopsy, the government agency Statistics Sweden identified up to five controls from the population matched for age, sex, calendar period of birth and county of residence. For example, a girl living in the county of Blekinge, diagnosed with CD in 2006 at the age of 13 years; was matched with five 13-year-old girls who were living in Blekinge in 2006. After exclusion of individuals with data irregularities, [18] we identified 228,632 controls (Figure 1).

Individuals undergoing biopsy and their matched controls were then linked to the Swedish Prescribed Drug Register (established on July 1st 2005) [19]. Through this linkage, we identified individuals biopsied between July 1st 2005 and January 29th 2008 (end of the study period). Thus, the final analyses included 2,933 individuals with CD, 2,118 individuals with inflammation, 620 individuals with normal mucosa but positive CD serology and 28,262 controls (Figure 1).

### Antibiotic use

The Swedish Prescribed Drug Register contains prospectively recorded individual data (on e.g. date of dispensing) on more than 99% of all dispensed prescribed drugs in Sweden [19]. Antibiotics in Sweden are not sold over the counter.

We collected data on use of all systemic antibiotics (anatomical therapeutic chemical, ATC code: J01) from July 1st 2005 (launch of the Prescribed Drug Register) through January 29th 2008 (end of the study period), and up to the date of the biopsy (and the corresponding date in matched controls). Antibiotics were grouped into penicillin V, extended-spectrum penicillins, quinolones, macrolides and other systemic antibiotics (Additional file 1).

### Statistical analyses

We used conditional logistic regression to estimate odds ratios (ORs) and 95% confidence intervals (CIs). Each stratum (one individual undergoing biopsy and up to five matched controls) was analyzed separately before a summary OR was calculated.

In our main analysis we examined the association between use of any systemic antibiotics and subsequent CD. Early-onset CD (i.e. before the age of 2 years) may have different risk factors compared with late-onset CD [5]. Additionally, antibiotic exposure early in life may have a more profound impact on the composition of the microbiota [20]. Accordingly, we performed stratified analyses by age at CD diagnosis (<2 years, 2–19 years, 20–39 years, 40–59 years and ≥60 years). We also stratified our analyses for sex. Similar sub-analyses were performed for individuals with small-intestinal inflammation and individuals with normal small-intestinal mucosa but positive CD serology. For each of these stratifications we examined for interaction via the inclusion in our models of multiplicative interaction terms, and the use of likelihood ratio tests between models with and without them.

Antibiotics differ in their influence on the intestinal microbiota. In pre-planned sub-analyses we estimated the association between CD and type of antibiotic exposure: penicillin V, extended-spectrum penicillins, quinolones, macrolides and other systemic antibiotics. This grouping of antibiotics has previously been used [21,22] and is largely based on the ATC classification system where the subgroups indicate the different therapeutic indications of antibacterial agents. To evaluate potential causality we estimated the dose- and time-dependent association between antibiotic use and CD in two separate analyses: (1) when individuals had received 1–2 courses and at least 3 courses of antibiotics and (2) when antibiotics had been prescribed in the year (≤365 days) before biopsy.

Education level has been associated with antibiotic use [23] and may influence the risk of CD diagnosis [24]. In a sub-analysis we therefore adjusted for education using seven predefined education categories determined by Statistics Sweden.

### Post-hoc analyses

Certain antiparasitic medications have similar pharmacokinetic and pharmacodynamic properties as systemic antibiotics with a strong impact on the gut microbiota. In a post-hoc analysis we therefore examined the relationship between use of any antiparasitic medications (ATC codes P01-P03, e.g. oral tinidazole) and CD, as well as specifically the use of metronidazole and CD.

Individuals with undiagnosed CD have an increased risk of comorbidity, [25] and with that, potentially increased surveillance and probability to receive antibiotic treatment. In a post-hoc analysis we therefore restricted our data to individuals who had not been admitted to a hospital during the study period. Hospital admission data were collected from the national Inpatient Register [26].

To further reduce the risk of surveillance bias we constructed a variable representing outpatient health care consumption. Hospital-based outpatient care has been recorded nationally in Sweden since January 1st 2001. We calculated the number of hospital-based outpatient visits from birth or start of the registry (whichever occurred latest) until the day before small-intestinal biopsy (or corresponding date in matched controls). We excluded visits in which CD was coded as the main reason for the visit. Individuals then were divided into four groups according to number of visits per year (those with no record of prior hospital-based outpatient care (0); >0 but <1 visit/year; 1- < 2 visits/year; and ≥2 visits/year). Those individuals with no record of hospital-based outpatient care may have undergone initial CD investigation in primary care before undergoing biopsy. For example: A patient A, undergoing biopsy in December 2006, with eight hospital-based outpatient visits in the six years between 2001 (start of registration of outpatient data) and December 2006 (time of biopsy) has an average of 1.3 visits per year (= 8 visits/6 years). In a post-hoc analysis, we added this variable to our statistical model to evaluate whether the association between antibiotic exposure and CD remained.

CD is elicited by dietary gluten and thus virtually nonexistent before the age of six months. To establish whether antibiotic use truly preceded CD, i.e. to evaluate the risk of reverse causation, we performed a sub-analysis of individuals who were exposed to antibiotics before the age of six months. In an additional post-hoc analysis we limited our exposure to antibiotic more than one year (>365 days) before CD diagnosis.

SPSS version 20.0 was used for all statistical analyses.

### Ethics

This study was conducted in accordance with the national and institutional standards and was approved by the Regional Ethical Vetting Board in Stockholm.

## Results

The median age at CD diagnosis in this study was 28 years. About 40% of those with CD were diagnosed in childhood and the majority of study participants were female (Table 1).

**Table 1:** **Descriptive characteristics of individuals with coeliac disease, small-intestinal inflammation, and normal small-intestinal mucosa**^**a**^

	**Coeliac disease**	**Inflammation**	**Normal mucosa**^**a**^
Total	2933	2118	620
Females (%)	1796 (61.2)	1336 (63.1)	396 (63.9)
Males (%)	1137 (38.8)	782 (36.9)	224 (36.1)
Age at study entry years (median; range)	28; 0-94	43; 0-98	36; 0-84
Age 0–19 (%)	1218 (41.5)	225 (10.6)	150 (24.2)
Age 20–39 (%)	566 (19.3)	684 (32.3)	202 (32.6)
Age 40–59 (%)	583 (19.9)	661 (31.2)	164 (26.5)
Age 60+ (%)	566 (19.3)	548 (25.9)	104 (16.8)
2005^b^ (%)	819 (27.9)	419 (19.8)	149 (24.0)
2006 (%)	1828 (62.3)	1074 (50.7)	304 (49.0)
2007 ^C^ (%)	274 (9.3)	582 (27.5)	167 (26.9)
2008 ^D^ (%)	12 (0.4)	43 (2.0)	-

^a^ Positive coeliac disease serology (IgA/IgG endomysial, tissue transglutaminase and antigliadin antibodies) 180 days before biopsy and until 30 days after biopsy in individuals with normal mucosa. Endomysial and tissue transglutaminase antibodies [IgA]: n = 139; Antigliadin antibodies [IgA/IgG] and endomysial and tissue transglutaminase antibodies [IgG]: n = 481.

^b^ Beginning of study period: July 1st 2005.

^c^ The majority of the pathology departments delivered data on individuals with small-intestinal pathology undergoing biopsy up to the beginning of year 2007. The remaining pathology departments reported histopathology data to the end of 2007 or very early 2008. For this reason, our data included fewer individuals with CD diagnosed in 2007 compared with 2006.

^D^ End of study period: January 29th 2008.

Reference individuals have not been included in the table because their age, sex and entry year distributions were identical to those of the individuals undergoing biopsy (due to matching).

Of the 2,933 individuals with CD, 27.0% had received at least one course of antibiotics during the study period before biopsy as compared with 21.1% in the controls, corresponding to an odds ratio for subsequent CD of 1.40 (95% CI = 1.27-1.53) (Table 2). In the individuals with inflammation but no villous atrophy 39.5% had used antibiotics as compared with 25.7% in the controls (OR = 1.90; 95% CI = 1.72–2.10). Antibiotic use also was associated with having a normal small-intestinal mucosa but positive CD serology (Table 2). Restricting our analysis to individuals with normal mucosa and positive IgA EMA or TTG did not influence the OR (Additional file 2). Adjustment for education level revealed unchanged ORs in all three groups (Additional file 2).

**Table 2:** **Odds ratios for prior antibiotic use in individuals with coeliac disease, small-intestinal inflammation and normal mucosa**^**a**^

	**Coeliac disease**				**Inflammation**				**Normal mucosa**^**a**^			
	**Cases**	**Controls**	**Odds ratio**	**95% CI**	**Cases**	**Controls**	**Odds ratio**	**95% CI**	**Cases**	**Controls**	**Odds ratio**	**95% CI**
	**(%)**	**(%)**			**(%)**	**(%)**			**(%)**	**(%)**		
**Any antibiotics**^**b**^	793/2933 (27.0)	3081/14571 (21.1)	1.40	1.27-1.53	836/2118 (39.5)	2687/10442 (25.7)	1.90	1.72–2.10	205/620 (33.1)	757/3069 (24.7)	1.58	1.30–1.92
**Courses of antibiotics**												
1-2 courses	639/2779 (23.0)	2573/14063 (18.3)	1.36	1.23-1.50	619/1901 (32.6)	2146/9901 (21.7)	1.75	1.57-1.95	153/568 (26.9)	621/2933 (21.2)	1.45	1.17-1.80
≥3 courses	154/2294 (6.7)	508/11998 (4.2)	1.58	1.31-1.92	217/1499 (14.5)	541/8296 (6.5)	2.50	2.10-2.97	52/467 (11.1)	136/2448 (5.6)	2.28	1.56-3.33
**Sex**												
Males	278/1137 (24.5)	1026/5645 (18.2)	1.48	1.27-1.72	282/782 (36.1)	824/3848 (21.4)	2.10	1.78-2.48	69/224 (30.8)	217/1099 (19.7)	1.93	1.38–2.69
Females	515/1796 (28.7)	2055/8926 (23.0)	1.36	1.21-1.52	554/1336 (41.5)	1863/6594 (28.3)	1.81	1.60-2.04	136/396 (34.3)	540/1970 (27.4)	1.43	1.12–1.82

Odds ratios estimated through conditional logistic regression modelling.

^a^ Positive coeliac disease serology 180 days before biopsy and until 30 days after biopsy in individuals with normal mucosa.

^b^ Antibiotics used between July 1st 2005 and January 29th 2008.

We found increasing ORs for repeated use of antibiotics and subsequent CD diagnosis (1–2 courses of antibiotics: OR = 1.36, 95% CI = 1.23-1.50; ≥3 courses of antibiotics: OR = 1.58, 95% CI = 1.31 - 1.92). Also in individuals with a biopsy showing inflammation or normal mucosa, but with positive CD serology, we found increasing ORs for repeated use of antibiotics, indicating a dose–response effect (Table 2).

The association between antibiotic treatment and subsequent CD was similar in males and females (Males: OR = 1.48, 95% CI = 1.27-1.72; Females: OR = 1.36; 95% CI = 1.21-1.52; p-value for interaction: 0.38). The stratified analyses by sex for individuals with small-intestinal inflammation and individuals with normal mucosa but positive CD serology are also presented in Table 2 (p-value for interaction, inflammation: 0.16; normal mucosa: 0.15). The stratified analyses by age at biopsy revealed only small differences between age groups and ORs for antibiotic treatment and development of CD, small-intestinal inflammation or normal mucosa but positive CD serology (Additional file 3). ORs for previous antibiotic treatment did not differ appreciably according to year of CD diagnosis (Additional file 3).

Overall, penicillin V was the most frequently prescribed antimicrobials, being used by 9.0% of the controls and nearly 10% of those with CD. Use of penicillin V was not associated with CD (OR = 1.12; 95% = 0.98 – 1.27). However, we found an association between use of each of the remaining types of antibiotic and subsequent CD, with essentially similar ORs, irrespective of antibiotic type (Table 3).

**Table 3:** **Odds ratios for prior antibiotic **^**a **^**use in individuals with coeliac disease**

	**Coeliac disease**			
	**Cases**	**Controls**	**Odds ratio**	**95% CI**
	**n = 2,933 (%)**	**n = 14,571 (%)**		
**Type of antibiotics used **^**b**^				
Penicillin V	291 (9.9)	1308 (9.0)	1.12	0.98 – 1.27
Extended spectrum penicillins	183 (6.2)	657 (4.5)	1.38	1.18 – 1.63
Quinolones	51 (1.7)	170 (1.2)	1.46	1.08 – 1.97
Macrolides	53 (1.8)	180 (1.2)	1.44	1.07 – 1.93
Other systemic antibiotics	291 (9.9)	1041 (7.1)	1.42	1.24 – 1.62
**Antibiotic use in the last year preceding diagnosis/study entry**				
Any antibiotic	722 (24.6)	2730 (18.7)	1.42	1.29 - 1.56
Penicillin V	259 (8.8)	1162 (8.0)	1.12	0.97 - 1.28
Extended spectrum penicillins	166 (5.7)	559 (3.8)	1.46	1.23 - 1.74
Quinolones	45 (1.5)	153 (1.1)	1.43	1.04 - 1.98
Macrolides	48 (1.6)	150 (1.0)	1.55	1.13 - 2.11
Other systemic antibiotics	206 (8.9)	905 (6.2)	1.47	1.26 - 1.66

Odds ratios estimated through conditional logistic regression modelling.

^a^ See Additional file 4 for anatomical therapeutic chemical codes used to classify systemic antibiotics (J01).

^b^ Antibiotics used between July 1st 2005 and January 29th 2008.

Twenty-five percent of the individuals with CD had received at least one course of antibiotics in the year before CD diagnosis compared with 18.7% of the matched controls (OR = 1.42; 95% CI = 1.29 - 1.56). ORs for type of antibiotic, according to ATC code, used in the year (≤365 days) before CD diagnosis are presented in Table 3.

### Post-hoc analyses

In a post-hoc analysis 115 individuals with CD (3.9%) and 259 controls (1.8%) had an earlier record of antiparasitic medication, equivalent to an OR of 2.12 for subsequent CD (95% CI = 1.72 - 2.62). Looking specifically at the earlier use of metronidazole revealed a slightly stronger association with CD (OR = 2.25; 95% CI = 1.71-2.96) (metronidazole use in the year before CD diagnosis: OR = 2.38; 95% CI = 1.78-3.19; and ≥ three courses of metronidazole: OR = 1.90; 95% CI = 0.62-5.78). Use of metronidazole was similarly associated with small-intestinal inflammation and normal mucosa but positive CD serology (Additional file 4).

To reduce the confounding effect of comorbidity we restricted our data to individuals with no hospital admissions (CD: n = 2,047; controls: n = 12,069). However, this post-hoc analysis revealed only a marginally changed OR for subsequent CD in relation to antibiotic use (OR = 1.30; 95% CI = 1.16 – 1.46). Post-hoc adjustment for number of outpatient visits before biopsy slightly changed the OR for CD (OR = 1.19; 95% CI = 1.08-1.31). Further, antibiotic use more than one year before biopsy examination was also associated with subsequent CD (OR = 1.30; 95% CI = 1.08-1.56).

We also estimated the OR for subsequent CD based on use of any antibiotics during the first six months of life. Only 3 of 16 (18.8%) children born after July 2005 and subsequently diagnosed with CD had been exposed to any antibiotics during their first six months of life, as compared with 7/80 (8.8%) children in the controls (OR = 2.26; 95% CI = 0.55-9.25).

## Discussion

This is the first study to find a positive association between antibiotic use and subsequent CD. Antibiotic exposure was also linked to small-intestinal inflammation and to normal mucosa with positive CD serology, both of which may represent early CD. The consistent association between the multiple groups, the slightly stronger association between repeated use of antibiotics compared with no use as well as the association with use of certain antibiotics (e.g., metronidazole) and CD may suggest that antibiotic exposure, possibly through a changed gut microbiota, plays a pathogenic role in early CD development. However, given the lack of time-response effect, within the limited time window studied, we cannot rule out non-causal explanations for our findings.

Observational studies on drugs are particularly susceptible to the concerns of reverse causation and confounding-by-indication. Reverse causation defines the causality bias if the exposure is a response to manifestations of the undiagnosed disease. In CD it is difficult to date the true onset of disease and thereby to establish whether antibiotic use truly preceded CD or whether the antibiotic was given for the symptoms of as yet undiagnosed CD. Several studies have shown a mean diagnostic delay of 5–11 years from onset of CD symptoms until diagnosis, [27] a time associated with an increased number of consultation visits [28] and possibly an increased likelihood of receiving antibiotic prescriptions. To reduce the risk of reverse causation and the effect of comorbidity, which may act as a confounder by increasing the possibility of receiving antibiotic prescriptions, we performed two post-hoc analyses restricted to individuals exposed to antibiotics in the first six months of life or individuals without hospital admission. Although these post-hoc analyses revealed largely unchanged ORs, they do not rule out residual comorbidity or reverse causation.

Observational studies on drugs may also be subject to confounding-by-indication in which the indication for treatment and not the treatment *per se* is associated with the outcome. Individuals with undiagnosed CD have an increased risk of several diseases that may, in concert, increase their likelihood to receive antibiotics [25]. For example, because antibiotics are frequently misused in viral infections, [29] confounding may be introduced when antibiotics are erroneously used to combat adenovirus or rotavirus infections, both proposed as risk factors for CD development [2]. However, the Swedish Medical Products Agency do not recommend antibiotic treatment in diarrhoeal illnesses, except for cases of severe bacterial gastroenteritis [30]. Further, just as for *diagnosed* CD, undiagnosed CD may be associated with bacterial infections, [31] which may have also influenced our results. Finally, the fact that all three cohorts were similarly associated with antibiotic use raises the possibility that an external factor, i.e. gastrointestinal symptoms such as diarrhoea, increases the “risk” of both antibiotic use and the performance of a small bowel biopsy.

It is well-established that the intestinal microbiota influences the maturation of the intestinal immune system [32]. Meanwhile several studies have found an imbalanced composition of the intestinal microbiota in those with CD [33]. *In vitro* studies suggest that intestinal dysbiosis may, in the presence of gliadin, increase intestinal epithelial permeability [10] and enable epithelial translocation of gliadin peptides potentially triggering CD [2]. Other data suggest that the distinct intestinal microbiota in CD may have pro-inflammatory properties that affect the immune response elicited by gluten [34]. Although this study lacks conclusive evidence for a *causal* association between antibiotic use and subsequent CD, our results do not refute the hypothesis that the intestinal microbiota affects CD development. A causal association may also be supported by the slightly stronger association to subsequent CD and certain antibiotics (e.g., metronidazole) that have a major impact on the anaerobic bacteria of the colon. Consequently, today’s prevalent use of antibiotics and their potential public heath impact on CD development warrant attention in future research.

Antibiotic use has been associated with the development of several immunological diseases, including inflammatory bowel disease [35] and asthma [36]. More importantly with regard to CD, most [22,37] but not all studies, [38] have failed to find an association between antibiotic use and subsequent type 1 diabetes, a disease that otherwise shares many aetiological traits with CD [39].

A major strength of this study is our use of multiple groups on the CD spectrum (CD, small-intestinal inflammation and normal mucosa with positive CD serology) [18]. With this study design, we were able to examine the association of antibiotic treatment by the degree of mucosal abnormality. Multiple groups also improved our evaluation of potential causality. Another strength is the use of prospectively recorded exposure and outcome data, which eliminate the risk of recall bias. Furthermore, this study provided detailed information on antibiotic use, including time and age of exposure, type of antibiotics and number of courses.

The use of biopsy data enabled us to identify a representative population with CD. In Sweden, more than 95% of gastroenterologists obtain a small-intestinal biopsy before CD diagnosis [14], implying that biopsy records have a high sensitivity for diagnosed CD. We regard the risk of misclassification in CD as low. In an earlier validation study 108 (95%) of 114 individuals with villous atrophy had CD [14]. Misclassification could be more of a concern in inflammation because villous atrophy may be patchy and not all inflammation is related to CD or to a pre-coeliac state. Furthermore, any potential misclassification of histopathology should be non-differential regarding antibiotic use and therefore should not lead to spurious associations, but to an underestimation of the true effect.

Our third cohort included individuals with normal small-intestinal mucosa, but positive CD serology. Most of these individuals had a single positive AGA serology with a lower specificity for CD than TTG or EMA. Thus, it may be argued that this condition does not represent a pre-coeliac state. However, when Hill *et al.* reviewed 26 studies of CD serology, they observed a median AGA specificity of 93% [40].

Antibiotic exposure was determined by the Swedish Prescribed Drug Register, which includes nationwide high-quality data on all dispensed prescribed medications [19]. Self-medication, i.e. obtaining an antibiotic without prescription, is very rare in Sweden, estimated to be 0.3% of all antibiotics used [41]. A limitation of our study is the recent start of the Swedish Prescribed Drug Register (established in July 2005) and the left truncation of exposure data in which individuals diagnosed with CD early in the study period (and their matched controls) will have little chance of being classified as antibiotic users because of lack of antibiotic data before July 2005. However, this loss of prior antibiotic data should not be differentially related to future CD status, and therefore only bias our results toward the null.

## Conclusions

In conclusion, we found a positive association between antibiotic use and subsequent CD, as well as with inflammation, and with having a normal mucosa but positive CD serology. One explanation could be that antibiotic exposure, possibly through changes in the gut microbiota, plays a role in early CD development, but non-causal explanations cannot be ruled out. Within the limited time window studied, the lack of a time-response effect raises the possibility of reverse causation, in particular, prescription of antibiotics to individuals with manifestations of undiagnosed CD.

## Abbreviations

ATC: Anatomical therapeutic chemical (pharmaceutical classification); AGA: Antigliadin antibody; CD: Coeliac disease; CI: Confidence interval; EMA: Endomysial antibody; OR: Odds ratio; TTG: Tissue transglutaminase antibody.

## Competing interests

The authors declare that they have no competing interests.

## Authors’ contributions

ICMJE criteria for authorship read and met: KM, WY; BL; PG; MB; TC; JFL. Agree with the manuscript’s results and conclusions: KM, WY; BL; PG; MB; TC; JFL. Designed the experiments/the study: KM, JFL. Collected data: JFL. Analyzed the data: KM. Wrote the first draft of the paper: KM. Contributed to the writing of the paper: WY; BL; PG; MB; TC; JFL. Contributed to the design of the study and interpretation of the data analyses: WY; BL; PG; MB; TC. Interpretation of data; approved the final version of the manuscript: KM, WY; BL; PG; MB; TC; JFL. Responsible for data integrity: KM, JFL. Supervised the project including data analyses: JFL. Obtained funding: JFL. All auhtors read and approved the final manuscript.

## Pre-publication history

The pre-publication history for this paper can be accessed here:

http://www.biomedcentral.com/1471-230X/13/109/prepub

## Supplementary Material

Additional file 1 Anatomical therapeutic chemical codes used to classify systemic antibiotics (J01). Click here for file

Additional file 2 **Odds ratios for prior antibiotic use in individuals with normal mucosa and positive coeliac disease serology.** Odds ratios (ORs) for prior antibiotic use with adjustment for education level. Click here for file

Additional file 3 **Odds ratios for prior antibiotic use in individuals with coeliac disease, small-intestinal inflammation, and normal small-intestinal mucosa**^**a**^**.** Stratified analyses by age at biopsy. Odds ratios for prior antibiotic use in individuals with coeliac disease. Stratified analyses by year of diagnosis. Click here for file

Additional file 4 **Odds ratio for prior use of metronidazole in individuals with small-intestinal inflammation and normal mucosa**^**a**^**.** Click here for file
