# Supplementary material for: Antibiotic exposure and the development of coeliac disease: a nationwide case–control study
Source: BMC Gastroenterol. 2013 Jul 8;13:109. doi: 10.1186/1471-230X-13-109 (PMC3720284; doi:10.1186/1471-230X-13-109)
Supplement: Additional file 1 — Anatomical therapeutic chemical codes used to classify systemic antibiotics (J01). [file 1471-230X-13-109-S1.pdf]

Additional file 1. Anatomical therapeutic chemical codes used to classify systemic antibiotics (J01).

Type of systemic antibiotics	Anatomical therapeutic chemical code	Description
Penicillin V	J01CE	Beta-lactamase sensitive penicillins
Extended spectrum penicillins	J01CA	Penicillins with extended spectrum
	J01CF	Beta-lactamase resistant penicillins
	J01CG	Beta-lactamase inhibitors
	J01CR	Combinations of penicillins
Quinolones	J01M	Quinolone antibacterials
Macrolides	J01F	Macrolides, lincosamides and streptogramins
Other systemic antibiotics	J01A	Tetracyclines
	J01B	Amphenicols
	J01D	Other beta-lactam antibacterials
	J01E	Sulfonamides and trimethoprim
	J01G	Aminoglycoside antibacterials
	J01R	Combinations of antibacterials
	J01X	Other antibacterials
