# Supplementary material for: Antibiotic exposure and the development of coeliac disease: a nationwide case–control study
Source: BMC Gastroenterol. 2013 Jul 8;13:109. doi: 10.1186/1471-230X-13-109 (PMC3720284; doi:10.1186/1471-230X-13-109)
Supplement: Additional file 2 — Odds ratios for prior antibiotic use in individuals with normal mucosa and positive coeliac disease serology. Odds ratios (ORs) for prior antibiotic use with adjustment for education level. [file 1471-230X-13-109-S2.pdf]

Additional file 2.

Odds ratios for prior antibiotic use in individuals with normal mucosa and positive coeliac disease serology [a]

	**Normal mucosa [a]**			
	Cases n = 139 (%)	Controls n = 819 (%)	Odds ratio	95% CI
**Any antibiotics[b]**	54 (38.8)	225 (27.5)	1.73	1.17 – 2.56

[a] Positive IgA Endomysial or tissue transglutaminase test 180 days before biopsy and until 30 days after biopsy in individuals with normal mucosa.

[b] Antibiotics used between July 1st 2005 and January 29th 2008.

Odds ratios (ORs) for prior antibiotic use with adjustment for education level:

Coeliac disease: OR = 1.42; 95% CI = 1.29-1.56; Inflammation: OR = 1.90; 95% CI =1.72-2.11; Normal mucosa with positive coeliac disease serology: OR = 1.59; 95% CI = 1.31-1.94.
