# Supplementary material for: Antibiotic exposure and the development of coeliac disease: a nationwide case–control study
Source: BMC Gastroenterol. 2013 Jul 8;13:109. doi: 10.1186/1471-230X-13-109 (PMC3720284; doi:10.1186/1471-230X-13-109)
Supplement: Additional file 3 — Odds ratios for prior antibiotic use in individuals with coeliac disease, small-intestinal inflammation, and normal small-intestinal mucosaa. Stratified analyses by age at biopsy. Odds ratios for prior antibiotic use in individuals with coeliac disease. Stratified analyses by year of diagnosis. [file 1471-230X-13-109-S3.pdf]

Additional file 3. Odds ratios for prior antibiotic use in individuals with coeliac disease, small-intestinal inflammation, and normal small-intestinal mucosa [a]. Stratified analyses by age at biopsy.

Age	Coeliac disease				Inflammation				Normal mucosa [a]			
< 2 years	51/132 (38.6)	189/655 (28.9)	1.58	1.07-2.34	6/12 (50.0)	15/60 (25.0)	4.72	0.85-26.17	5/17 (29.4)	28/85 (32.9)	0.84	0.26-2.69
2-19 years	274/1086 (25.2)	1055/5419 (19.5)	1.40	1.20-1.63	64/213 (30.0)	199/1060 (18.8)	1.92	1.37-2.69	41/133 (30.8)	187/665 (28.1)	1.15	0.76–1.76
20-39 years	149/566 (26.3)	612/2809 (21.8)	1.30	1.05-1.60	264/684 (38.6)	904/3371 (26.8)	1.72	1.45-2.05	60/202 (29.7)	216/1004 (21.5)	1.62	1.14–2.31
40-59 years	148/583 (25.4)	558/2905 (19.2)	1.45	1.18-1.80	267/661 (40.4)	863/3279 (26.3)	1.91	1.60-2.27	57/164 (34.8)	187/812 (23.0)	1.91	1.30–2.79
60+ years	171/566 (30.2)	667/2783 (24.0)	1.40	1.14-1.72	235/548 (42.9)	706/2672 (26.4)	2.13	1.75-2.58	42/104 (40.4)	139/503 (27.6)	1.93	1.22–3.07

Odds ratios estimated through conditional logistic regression modelling.

[a] Positive coeliac disease serology 180 days before biopsy and until 30 days after biopsy in individuals with normal mucosa.

Odds ratios for prior antibiotic use in individuals with coeliac disease. Stratified analyses by year of diagnosis.

**Year of diagnosis**	**Coeliac disease** Cases (%)	Controls (%)	Odds ratio	95% CI
2005	98/819 (12.0)	311/4,062 (7.7)	1,62	1,28-2,05
2006	550/1,828 (30.1)	2,180/9,097 (24.0)	1,35	1,21-1,51
2007-2008	145/286 (50.7%)	590/1412 (41.8%)	1.42	1,10-1,83

Odds ratios estimated through conditional logistic regression modelling.
