# Supplementary material for: Antibiotic exposure and the development of coeliac disease: a nationwide case–control study
Source: BMC Gastroenterol. 2013 Jul 8;13:109. doi: 10.1186/1471-230X-13-109 (PMC3720284; doi:10.1186/1471-230X-13-109)
Supplement: Additional file 4 — Odds ratio for prior use of metronidazole in individuals with small-intestinal inflammation and normal mucosaa. [file 1471-230X-13-109-S4.pdf]

Additional file 4.
Odds ratio for prior use of metronidazole in individuals with small-intestinal inflammation and normal mucosa[a].

	Inflammation				Normal mucosa [a]			
	Cases (%)	Controls (%)	Odds ratio	95% CI	Cases (%)	Controls (%)	Odds ratio	95% CI
**Metronidazole[b]**	126/2118 (5.9)	189/10,442 (1.8)	2.93	2.38-3.60	26/620 (4.2)	41/3069 (1.3)	2.98	1.86–4.76

Odds ratios estimated through conditional logistic regression modelling.

[a] Positive coeliac disease serology 180 days before biopsy and until 30 days after biopsy in individuals with normal mucosa.

[b] Any use of metronidazole between July 1st 2005 and January 29th 2008.
